# Early prediction of preeclampsia and small-for-gestational-age via multi-marker model in Chinese pregnancies: a prospective screening study

**DOI:** 10.1186/s12884-019-2455-8

**Published:** 2019-08-19

**Authors:** Jing Zhang, Luhao Han, Wei Li, Qiaobin Chen, Jie Lei, Min Long, Weibin Yang, Wenya Li, Lizhen Zeng, Sifan Zeng

**Affiliations:** Department of Clinical Laboratory, Shenzhen Nanshan Maternity and Child Healthcare Hospital, No.1 Wanxia Road, Nanshan District, Shenzhen, Guangdong People’s Republic of China

**Keywords:** Chinese, First-trimester screening, Preeclampsia, Small-for-gestational-age, Risk prediction

## Abstract

**Background:**

Recent evidence suggests early screening of preeclampsia and small-for-gestational-age (SGA) would benefit pregnancies followed by subsequent prophylactic use of aspirin. Multi-marker models have shown capability of predicting preeclampsia and SGA in first trimester. Yet the clinical feasibility of combined screening model for Chinese pregnancies has not been fully assessed. The aim of this study is to evaluate the applicability of a multi-marker screening model to the prediction of preeclampsia and SGA in first trimester particularly among Chinese population.

**Methods:**

Three thousand two hundred seventy pregnancies meeting the inclusion criteria took first-trimester screening of preeclampsia and SGA. A prior risk based on maternal characteristics was evaluated, and a posterior risk was assessed by combining prior risk with multiple of median (MoM) values of mean arterial pressure (MAP), serum placental growth factor (PLGF) and pregnancy associated plasma protein A (PAPP-A). Both risks were calculated by Preeclampsia PREDICTOR™ software, Perkin Elmer. Screening performance of prior and posterior risks for early and late preeclampsia by using PREDICTOR software was shown by Receiver Operating Characteristics (ROC) curves. The estimation of detection rates and false positive rates of delivery with both preeclampsia and SGA was made.

**Results:**

Eight cases developed early preeclampsia (0.24%) and 35 were diagnosed as late preeclampsia (1.07%). Five with early preeclampsia and ten with late preeclampsia later delivered SGA newborns (0.46%); 84 without preeclampsia gave birth to the SGAs (2.57%). According to ROC curves, posterior risks performed better than prior risks in terms of preeclampsia, especially in early preeclampsia. At 10% false positive rate, detection rates of early and late preeclampsia were 87.50 and 48.57%, detection rates of early and late SGA were 41.67 and 28.00%, respectively. For SGA, detection rates in cases with preeclampsia were much higher than those in absence of it.

**Conclusions:**

This study demonstrates that combined screening model could be useful for predicting early preeclampsia in Chinese pregnancies. Furthermore, the performance of SGA screening by same protocol is strongly associated with preeclampsia.

**Electronic supplementary material:**

The online version of this article (10.1186/s12884-019-2455-8) contains supplementary material, which is available to authorized users.

## Background

Preeclampsia (PE) and small -for -gestational -age (SGA) are two of the main contributors to maternal and perinatal morbidity and mortality worldwide [1–3], which could impose life-long negative effects on an individual [4, 5]. PE is a disorder of pregnancy with typical characteristics of hypertension and proteinuria, which affects multiple physiological systems after 20 weeks’ gestation [6]; SGA newborns are generally defined as those with weight below the 10th percentile for the gestational age [7]. Early identification of PE and SGA will enable clinicians to take preventative steps and offer intensive monitoring to the high-risk pregnancy group, thereby decreasing the incidence of serious diseases in both mothers and their offspring.

According to the guidelines of the UK National Institute for Health and Clinical Excellence (NICE) and the American Congress of Obstetricians and Gynecologists (ACOG), the traditional approach to evaluate women with high risk of developing PE is to derive risk factors from their medical histories and maternal demographic features. This kind of screening method remains recommended for clinical use [8, 9]. Previous studies have demonstrated that maternal serum pregnancy associated plasma protein A (PAPP-A) and placental growth factor (PLGF) MoM levels at 11 to 13 weeks’ gestation were lowered in pregnant women who subsequently developed PE and also in those who delivered SGA infants [10, 11]. In recent years, the Fetal Medicine Foundation (FMF) has developed a new algorithm for screening high-risk PE pregnancies at 11–13 weeks’ gestation, combining a number of maternal factors such as mean arterial pressure (MAP), uterine artery pulsatility index (UTPI), serum PLGF and PAPP-A MoM values [12, 13]. It outperforms the existing criteria recommended by NICE and ACOG [14, 15]. The algorithm has also shown potential to predict delivery of SGA [16], since the two placenta-related pregnancy complications (PE and SGA) may share the common pathophysiological mechanism. Moreover, the FMF screening algorithm has already been widely validated in Europe [17–19].

Heretofore, most of the combined model studies on prediction of PE and SGA have been focused on the Caucasian and African-American populations, whereas the data of the Asians are insufficient. Our study adopted the algorithm in Pre-Eclampsia Predictor™ by Perkin Elmer (hereafter named “PREDICTOR”) which has similar performance to the FMF algorithm in predicting PE [20]. It is a standalone software application designed for calculating prior and posterior risks of early and late PE in first trimester, and performs risk calculation by using the Bayesian algorithm developed by Professor Howard Cuckle [21]. No previous study has investigated the applicability of PREDICTOR algorithm to predicting PE and SGA by combining maternal factors and biomarkers in first trimester in Mainland China. More importantly, an effective screen method for PE and SGA will allow more accurate research on prophylactic intervention. In light of these considerations, it is significant to demonstrate the effectiveness of the PREDICTOR algorithm in prediction of PE and SGA in Mainland Chinese pregnancies.

This study aims to, firstly, estimate the predictive performance of screening model for PE in Chinese pregnant women in first trimester through a combination of maternal characteristics, MAP, serum PLGF and PAPP-A, by applying PREDICTOR algorithm; secondly, to investigate potential predictive value for SGA through the same screening protocol.

## Methods

### Subjects

From December 2016 to May 2018, pregnant women attending the first-trimester trisomy screening (11^+ 0^ to 13^+ 6^ weeks) at Shenzhen Nanshan Maternity and Child Healthcare Hospital were recruited in this prospective study. Exclusion criteria were as follows: (1) multiple pregnancies; (2) pregnancies with aneuploidies; (3) major fetal abnormalities; (4) women who experienced termination of pregnancy, miscarriage or stillbirth before 24 weeks. Those recruited gave informed consent in writing to participate in this research which had been duly approved by the Medical Ethics Committee of Shenzhen Nanshan Maternity and Child Healthcare Hospital, Shenzhen, the People’s Republic of China. Detailed demographic data including medical and obstetric histories were collected from the information system of Shenzhen Nanshan Maternity and Child Healthcare Hospital. Also, patients’ pregnancy outcomes were followed up via Shenzhen Maternity and Child Health Management Information Digital System.

### Measurement of MAP, maternal serum PLGF and PPAP-A

MAP is defined as the average arterial pressure during a single cardiac cycle, and is calculated by the following formula: MAP = 2/3 diastolic blood pressure + 1/3 systolic blood pressure. It was measured in accordance with a standardized protocol [22]. Blood pressure was taken by automatic blood pressure monitor (Omron HBP 9020, Omron Healthcare Inc., Japan) which were calibrated regularly. The measuring and recording were made by nurses who had received adequate training before this study. The pregnancies were in a sitting position and their legs were not crossed. Their arms were posed at the level of the heart, each properly fitted with a cuff. After a five-minute rest, blood pressure was measured from both arms simultaneously with a one-minute interval for both arms until variation between consecutive readings fell within 10 mmHg in systolic and 6 mmHg in diastolic blood pressure in both arms. And then we calculated the MAP of each arm by the average of the last two stable measurements, and took the arm with the highest final MAP for the subsequent analysis of results. Maternal serum samples were obtained from routine first-trimester aneuploidy screening, of which PAPP-A concentration was quantified as a part. The measurement was performed by time-resolved fluoroimmunoassay at the Auto DELFIA® analytical platform (Perkin Elmer Inc., Turku, Finland) using Auto DELFIA® PAPPA kit (Perkin Elmer Inc., Wallac Oy, Turku, Finland). Serum PLGF concentration was measured by the same device of Auto DELFIA® PLGF 1-2-3 kits (Perkin Elmer Inc., Wallac Oy, Turku, Finland).

### Definitions

For this study, PE was defined as new onset of hypertension (≥140 mmHg systolic blood pressure and/or ≥ 90 mmHg diastolic blood pressure on ≥2 separate occasions 4 h apart) occurred after 20 weeks’ gestation together with proteinuria (≥300 mg/24 h collection of urine or urinary protein to creatinine ratio of ≥30 mg/mmol or two readings of at least + on dipstick analysis of a midstream or catheter urine specimen) in a previously normotensive patient [23]. Based on gestational age, PE at delivery was sub-categorized into either early (delivery < 34 weeks gestation) or late (delivery ≥34 weeks) PE [24]. SGA was defined as birth weight < 10th percentile in Chinese population [25]. A cutoff of gestational age at birth of 34 weeks was adopted to classify early and late SGA as suggested in PREDICOTR algorithm for PE, while no authoritative definition has as yet been maintained for early and late SGA.

### Calculation of risks and statistical analysis

Four types of PE risks (prior risk early, prior risk late, posterior risk early and posterior risk late) were calculated through Pre-Eclampsia Predictor™ software, named “PREDICTOR” (Perkin Elmer, version 1.1). The methodology was similar to aneuploidy screening. The calculation workflow contains four steps: (1) Prior risk was derived from the calculation based on separate likelihoods of each of five risk factors (BMI, ethnicity, parity, history of PE and chronic hypertension); (2) Multiples of the median (MoM) were calculated for markers (MAP, PAPP-A, PLGF) and gestation days were used in MoM calculation. Additionally, MoM values of PAPP-A and PLGF were adjusted for weight, ethnicity and smoking status; (3) The calculation of the likelihoods of the given markers adopted the multivariate Gaussian distribution; (4) Final risk (posterior risk) was assessed by combining the prior risk likelihoods with marker likelihoods. The program default setting gives the cutoff of posterior risk 1:20.

Data analysis was conducted by using the statistical software package SPSS 25.0 (SPSS Inc., Chicago, I11, USA). Comparisons between groups were made by chi-square test or Fisher’s exact test used for categorical variables as well as Mann Whitney-U test used for continuous variables. *P* values of < 0.05 were considered statistically significant. The sample size analysis was carried out by using the R package software (Version 3.6.0). Performance of screening was further examined by receiver operating characteristic (ROC) curve analysis using MedCalc (MedCalc Software, Version 11.4.20, Mariakerke, Belgium). The test performance was estimated by detection rates (DRs), false positive rates (FPRs) and positive predictable values (PPVs).

## Results

### Screening population and outcomes

**Fig. 1:**
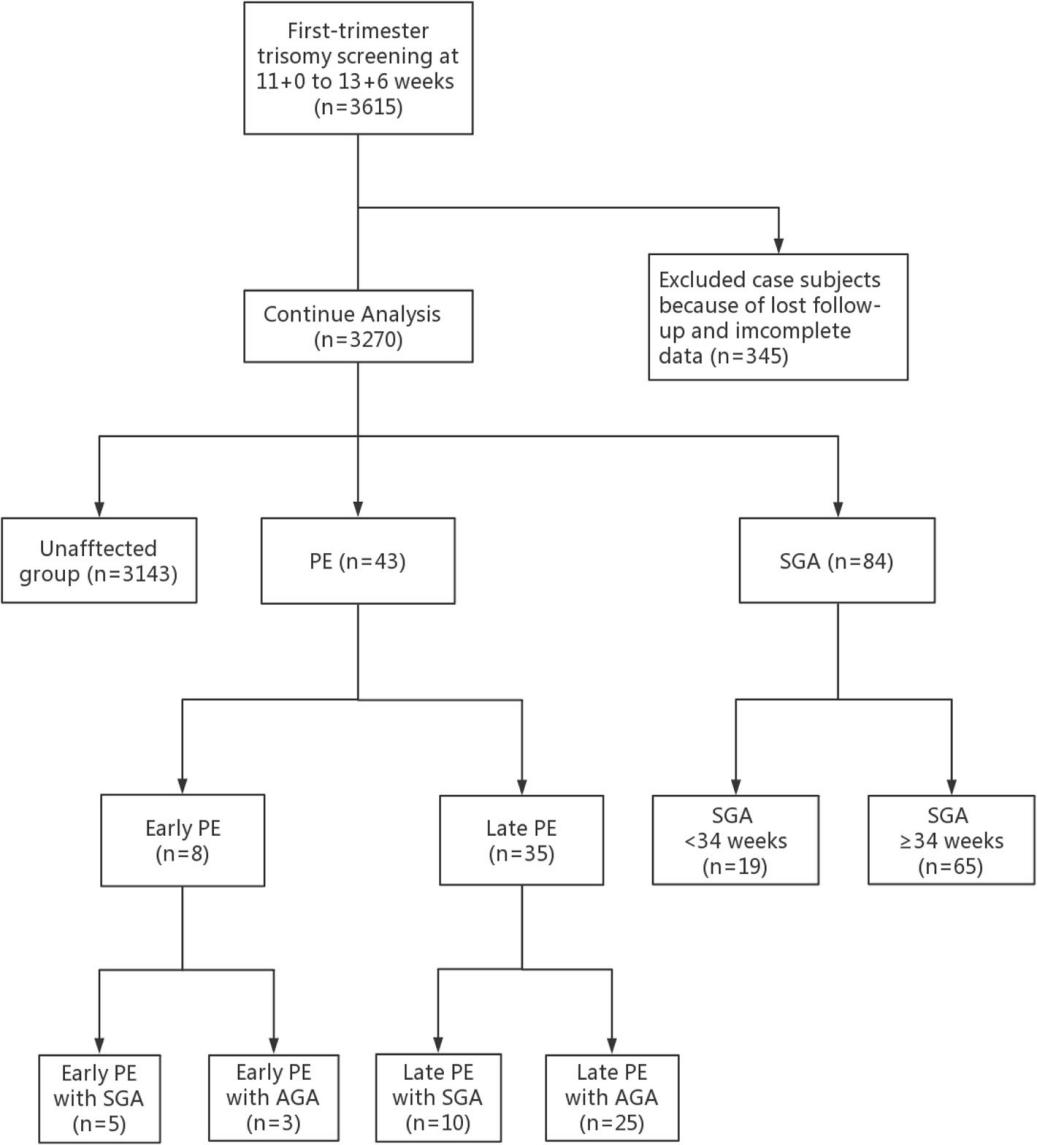
Flowchart of this prospective screening study. ***Legend*:** PE = Preeclampsia, SGA = Small-for-gestational-age, AGA = Appropriate-for-gestational-age, n = number

A total of 3615 pregnant women agreed to participate in this prospective study whereas 345 pregnancies were excluded due to either loss of follow-up or incomplete data. Thus, 3270 patients in total met the inclusion criteria in the final analysis. Among the recruited subjects, 43 women (1.31%) experienced PE including eight cases of early PE (0.24%) and 35 cases of late PE (1.07%). In the PE group, five out of eight pregnancies with early PE (0.15%) and ten out of 35 with late PE (0.31%) later delivered SGA newborns; 84 pregnancies (2.57%) in the absence of PE ultimately delivered SGA infants. A flow chart of this prospective study is depicted in Fig. 1.

### Characteristics of pregnancies

Participants were divided into Non-PE and PE groups, and the comparison of demographic and clinical characteristics is displayed in Table 1. It can be seen that maternal weight, weight gain during pregnancy, postpartum blood pressure, BMI and rate of chronic hypertension in women who later developed PE were higher than Non-PE group. Also, pregnancies in PE group experienced earlier delivery, and their babies’ birth weight were significantly lower than those in the unaffected group.

**Table 1:** Characteristics of pregnant women in PE and non-PE groups

Characteristics	Non-PE Group (*n* = 3227)	PE Group (*n* = 43)
Maternal Age, years	30 (28–34)	30 (28–34)
Paternal Age, years	32 (29–35)	31 (28–34)
Maternal Weight, kg	53.00 (48.80–58.60)	56.00 (51.50–65.00)*
Maternal Height, cm	160.00 (156.00–163.00)	157.75 (154.75–162.25)
CRL ^a^, mm	63 (59–68)	62 (56–67)
GA ^b^ at Screening, days	89 (86–91)	88 (85–91)
Smoking (%)	2 (0.06)	0
Spouse Smoking (%)	928 (28.76)	9 (20.93)
Drinking (%)	10 (0.31)	0
Spouse Drinking (%)	1079 (33.44)	11 (25.58)
BMI ^c^	20.90 (19.27–22.94)	23.28 (20.10–26.10)*
Conception (%)		
Spontaneous	3194 (98.98)	43 (100.00)
IVF ^d^	33 (1.02)	0 (0)
Medical History (%)		
Chronic Hypertension	19 (0.59)	8 (18.60)*
Diabetes Mellitus	10 (0.31)	0 (0)
Thyroid Disease	316 (9.79)	2 (4.65)
GA ^b^ at Delivery, weeks	39 (38–40)	36 (35–39)*
Pregnancy Weight Gain, kg	14.00 (11.00–16.65)	17.00 (13.50–18.59)*
Birth Weight, g	3270.00 (3010.00–3550.00)	2460.00 (1930.00–3030.00)*
Postpartum Blood Pressure, mmHg		
Systolic Blood Pressure	119 (112–125)	131 (123–144)*
Diastolic Blood Pressure	70 (65–76)	81 (72–92)*

Data are presented as median (interquartile range) or n (%). *P < 0.05

a: CRL = Crown rump length, b: GA = Gestation age, c: BMI=Body mass index, d: IVF=In vitro fertilization

### Levels of markers

The concentrations and MoM values of different markers in PE and SGA groups are depicted by median and interquartile range (IQR) in Table 2 (Original testing results of markers are shown in Additional File 1). The MoMs of MAP, serum PLGF and PAPP-A were statistically significantly different in the PE subgroups by comparison with the unaffected by PE group (*P* < 0.05). Additionally, serum PLFG and PAPP-A were remarkably lower in SGA group and SGA without PE group (P < 0.05) comparing with the unaffected by SGA group, respectively. However, there was no significant difference in MAP in the unaffected by SGA group and the SGA in absence of PE group.

**Table 2:** Multiple of the medians and concentrations for markers in different PE groups and SGA groups

	Serum PLGF, (Median, IQR)		Serum PAPP-A, (Median, IQR)		MAP, (Median, IQR)	
	MoM	CON, pg/mL	MoM	CON, mU/L	MoM	mmHg
PE Groups						
Unaffected by PE (n = 3227)	1.11 (0.79–1.47)	43.61 (30.71–58.18)	0.93 (0.67–1.29)	4074.56 (2764.41–5867.71)	0.97 (0.90–1.04)	78.83 (73.50–84.83)
PE (n = 43)	0.61 (0.43–0.87)**	23.93 (16.76–32.57)**	0.78 (0.42–1.17)**	2718.38 (1695.74–4276.59)**	1.06 (0.99–1.13)**	88.17 (81.17–93.00)**
Early PE (*n* = 8)	0.72 (0.49–0.90)**	25.31 (18.06–32.06)**	0.49 (0.28–1.15)*	1853.23 (847.76–3582.42)*	1.13 (1.10–1.18)**	96.17 (91.96–100.17)**
Late PE (*n* = 35)	0.59 (0.41–0.87)**	23.36 (16.76–33.20)**	0.83 (0.50–1.17)*	2932.82 (1900.94–4322.98)**	1.04 (0.99–1.12)**	85.33 (80.67–90.50)**
PE with SGA (*n* = 15)	0.61 (0.25–0.81)**	22.22 (9.40–29.47)**	0.54 (0.39–0.94)*	2216.32 (1226.38–4322.98)*	1.10 (1.00–1.14)**	90.67 (82.67–93.00)**
SGA Groups						
Unaffected by SGA (*n* = 3171)	1.10 (0.79–1.47)	43.62 (30.71–58.18)	0.93 (0.67–1.29)	4087.87 (2780.29–5863.93)	0.97 (0.90–1.04)	78.83 (73.50–85.00)
SGA (*n* = 99)	0.85 (0.58–1.26)**	32.50 (22.51–50.17)**	0.80 (0.49–1.20)**	3349.51 (2010.72–5188.12)**	1.01 (0.92–1.09)*	82.67 (74.29–88.87)*
SGA without PE (*n* = 84)	0.92 (0.63–1.28)**	36.59 (24.88–52.87)**	0.87 (0.52–1.20)*	3477.51 (2220.13–5284.75)*	0.98 (0.90–1.07)	79.93 (73.83–87.83)

Data derived from comparison of unaffected group with PE and SGA groups, respectively, by Mann-Whitney U test. Significance level: *p < 0.05; **p < 0.005. PE = Preeclampsia, SGA = Small-for-gestational-age, PAPP-A = Pregnancy associated plasma protein A, PLGF=Placental growth factor, MoM = Multiple of median, IQR = Interquartile range, CON=Concentration, n = number

### Screening performance of PREDICTOR algorithm

**Fig. 2:**
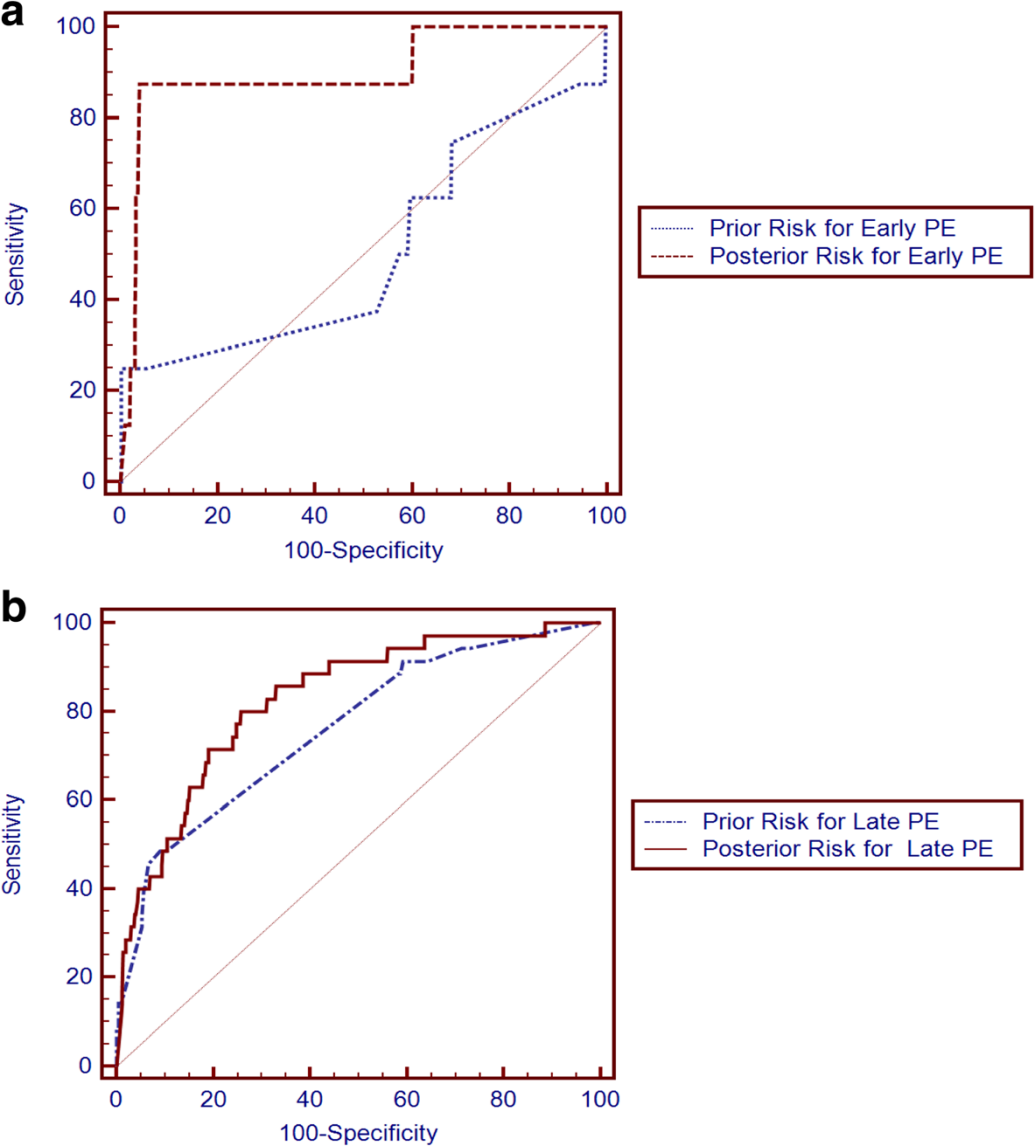
ROC curves with prediction model of prior and posterior risks for early and late PE. ***Legend:*** (**a**) (**∙∙∙∙∙∙**) Prior Risk for early PE, (**-----**) Posterior Risk for early PE; (**b**) (**−∙ − ∙ − ∙**) Prior Risk for late PE, (**——**) Posterior Risk for late PE

Using the PREDICTOR algorithm, both prior and posterior risks for early PE and late PE in the first trimester can be calculated separately. Receiver Operating Characteristics (ROC) curves for the screening performance of prior and posterior risks are presented in Fig. 2, and the Area Under the ROC (AUC) curves are given in Table 3. For early PE, the AUC curves were 0.509 (prior risk) and 0.901 (posterior risk); the AUC curves for late PE were 0.762 (prior risk) and 0.828 (posterior risk). By comparing prior risk with posterior risk, the latter outperformed the former, especially in predicting early PE (in comparison with two AUC curves, *P* < 0.05). It can be seen that the performance of predicting PE by using prior risk was fairly poor, therefore more concerns were put on the posterior risk.

**Table 3:** AUC curves of different PE risks with the best fit cut-off values

	Type of risk	AUC, 95%CI	cut-off value	DR%, 95CI	FPR%, 95CI
Early PE	Prior Risk	0.509 (0.491–0.526)	1:54	25 (3.2–65.1)	0.31 (0.1–0.6)
	Posterior Risk	0.901 (0.890–0.911)*	1:45	87.5 (47.3–99.7)	3.94 (3.3–4.7)
Late PE	Prior Risk	0.762 (0.747–0.776)	1:102	48.57 (31.4–66.0)	9.05 (8.1–10.1)
	Posterior Risk	0.828 (0.814–0.840)	1:151	80.00 (63.1–91.6)	25.81 (24.3–27.4)

AUC = Area under the receiver operating characteristics curve, DR = Detection rate, FPR = False positive rate, 95% CI = 95% Confidence interval. Cut-off values of different risks are at the highest average of sensitivity and specificity, and DRs and FPRs are shown at the best fit cut-off value of each type of risk. Pairwise comparison of ROC curves between prior risk and posterior risk in early PE and late PE individually, and significance level: * P < 0.05

The PE risk calculation software in this screening model defined high-risk PE pregnancy according to the default cutoff setting--posterior risk cutoff > 1:20 (e.g., 1:10). However, DRs at cutoff 1:20 were very low (25.00% in early PE, 34.29% in late PE). Yet, according to ROC curves, we obtained the cut-off values at the highest average of sensitivity and specificity (shown in Table 3), based on which we then found that the best posterior risk cut-offs were 1 in 45 for early PE (DR 87.50%, FPR 3.94%) and 1 in 151 for late PE (DR 80.00%, FPR 25.81%).

The DRs and positive predictive values (PPVs) of delivery with PE or SGA at < 34 weeks and ≥ 34 weeks under fixed FPRs of 5, 10 and 15% are demonstrated in Table 4. In this combined testing (at fixed FPRs), the DRs of early PE and early SGA were higher than those of late PE and late SGA. The DRs of early PE, early SGA, late PE, and late SGA were 87.50, 37.50, 40.00 and 20%, respectively, at FPR of 5%. With the exception of early PE, the DRs of other subgroups enhanced with increased FPR. When comparing PE + SGA group with PE + AGA (Appropriate-for-gestational-age) group, the DRs differed substantially (at 10% FPR, 100% versus 66.67% in early groups; 60.00% versus 44.00% in late groups).

**Table 4:** Performance of this combined screening at fixed FPRs in PE and SGA subgroups

	FPR 5%		FPR 10%		FPR 15%	
	DR %	PPV %	DR %	PPV %	DR %	PPV %
Posterior Risk Early (< 34 weeks)	**risk cut-off (1:64)**		**risk cut-off (1:172)**		**risk cut-off (1:293)**	
Early PE (n = 8)	87.50 (47.35–99.68)	4.17 (1.69–8.40)	87.50 (47.35–99.68)	2.12 (0.86–4.32)	87.50 (47.35–99.68)	1.43 (0.58–2.92)
Early PE with SGA (*n* = 5)	100.00 (47.82–100.00)	3.01 (0.99–6.89)	100.00 (47.82–100.00)	1.52 (0.50–3.52)	100.00 (47.82–100.00)	1.02 (0.33–2.37)
Early PE with AGA (n = 3)	66.67 (9.43–99.16)	3.11 (1.04–7.03)	66.67 (9.43–99.16)	1.58 (0.53–3.60)	66.67 (9.43–99.16)	1.06 (0.35–2.42)
Early SGA (*n* = 24)	37.50 (18.80–59.41)	5.36 (2.48–9.93)	41.67 (22.11–63.36)	3.06 (1.48–5.55)	45.83 (25.55–67.18)	2.26 (1.13–4.01)
Early SGA without PE (*n* = 19)	21.05 (6.05–45.57)	2.45 (0.67–6.16)	26.32 (9.15–51.20)	1.55 (0.51–3.59)	31.58 (12.58–56.55)	1.24 (0.46–2.69)
Posterior Risk Late (≥34 weeks)	**risk cut-off (1:25)**		**risk cut-off (1:48)**		**risk cut-off (1:75)**	
Late PE (*n* = 35)	40.00 (23.87–57.89)	8.00 (4.44–13.06)	48.57 (31.38–66.01)	5.00 (2.94–7.88)	60.00 (42.11–76.13)	4.16 (2.59–6.29)
Late PE with SGA (*n* = 10)	60.00 (26.24–87.84)	3.59 (1.33–7.66)	60.00 (26.24–87.84)	1.82 (0.67–3.93)	70.00 (34.75–93.33)	1.43 (0.58–2.92)
Late PE with AGA (*n* = 25)	32.00 (14.95–53.50)	1.52 (0.25–4.79)	44.00 (24.40–65.07)	4.54 (2.58–7.34)	56.00 (34.93–75.60)	3.88 (2.37–5.96)
Late SGA (*n* = 75)	20.00 (11.65–30.83)	8.62 (4.91–13.82)	28.00 (18.24–39.56)	6.21 (3.89–9.34)	40.00 (28.85–51.96)	5.93 (4.04–8.36)
Late SGA without PE (*n* = 65)	13.85 (6.53–24.66)	5.36 (2.48–9.93)	23.08 (13.53–35.19)	4.52 (2.55–7.34)	35.38 (23.92–48.23)	4.61 (2.94–6.84)

DR = Detection rate, FPR = False positive rate, PPV=Positive predictable value, 95% CI = 95% Confidence interval, PE = Preeclampsia, SGA = Small-for-gestational-age, AGA = Appropriate-for-gestational-age, n = number

## Discussion

This prospective study demonstrated that our screening model at 11–13 weeks’ gestation could identify pregnancies developing early PE efficiently; furthermore, it performed better in detection of PE with SGA than PE with AGA. Nevertheless, by using the same screening method, the performance of prediction of isolated early and late SGA was evidently lower. Thus, we supposed that the prediction of SGA may depend heavily on the presence of PE. Moreover, this study found that women with PE or those delivered SGA had decreased serum PLGF and PAPP-A. And previous studies have proposed that the low concentrations of serum markers in the first trimester are likely to be the consequence of impaired trophoblastic invasion of the spiral arteries [26]. Although the pathophysiological mechanisms behind PE and SGA are still not fully understood, impaired placenta indeed plays a central role in the development of these two conditions.

In this study, we used the PREDITCOR algorithm developed by Professor Howard Cuckle to calculate early and late PE risks. The prior risk is a product of a combination of multiple demographic-based likelihoods. In the aspect of diagnostic accuracy, it is less effective than using posterior risk (combining prior likelihoods and likelihoods of MAP, PLGF and PAPP-A). Our data provide further support for the hypothesis that screening performance for PE at 11–13 weeks’ gestation could be improved significantly by adding MAP, serum PLGF and PAPP-A. Furthermore, according to the aforesaid software default setting, the theoretic posterior risk cutoff (1:20) was less effective in early screening of PE and apparently not suitable for the population in our research, whereas from our calculation, the suitable posterior risk cutoffs for early and late PE were 1:45 and 1:151, individually. Therefore, we can draw a conclusion that laboratories should adjust the cutoff values in accordance with the screening population and their lab conditions in order to achieve the best screening performance to define high-risk PE group in the most appropriate fashion.

The past decade has seen extensive studies being conducted to screen for PE based on the FMF algorithm which combines maternal characteristics with different biochemical and biophysical markers at 11–13 weeks’ gestation [13, 17, 19]. Yet only a very few medical units applied the PREDICTOR algorithm to test its validity in prediction of PE and SGA in first trimester [20]. Moreover, in reviewing literature, few data has been found relating to predicting PE and SGA risks in Chinese pregnancies at 11–13 weeks’ gestation by using the PREDICTOR algorithm.

It is noteworthy that the findings in this report indicate that Perkin Elmer’s PREDICTOR algorithm is an effective method of detecting early PE in Chinese pregnancies. According to Table 4, the DRs of early PE, under 5 and 10% FPRs, were both 87.5%, which were similar to those of previous researches conducted in western countries. For instance, a prospective observational nonintervention cohort study in a U.S. population screened for PE through a combination of maternal characteristics, MAP, UTPI and serum biomarkers (PLGF, PAPP-A, alphafetoprotein), in which the DRs of early PE, at either 5% or 10% FPR, were both 85% [27]. Also, another previous study showed that among 8775 pregnancies, the DRs of delivery with PE < 32 weeks’ gestation screened by maternal factors combining MAP, PAPP-A and PLGF, at FPRs of 5 and 10%, were 76 and 88%, respectively [17]. Additionally, O’Gorman et al. screened 35,948 singleton pregnancies at 11 to 13 weeks among whom they found that by combining maternal factors with MAP, serum PLGF and PAPP-A in screening, the DRs of early PE delivery before 32 weeks’ gestation, at 5 and 10% FPRs, were 76 and 85%, respectively [28]. In our study, we concluded that DRs for SGA differed substantially with and without PE. A similar conclusion was made by a previous study, which assessed the effectiveness of first-trimester screening for SGA neonates using a combination of maternal characteristics, MAP, serum biochemical markers and UTPI [29]. The DR for early SGA in their study was higher and the DR for late SGA was lower than the data in our report (60% versus 41.67% in early, and 23% versus 28% in late, at FPR of 10%).

This study carries following strengths. First, it is a prospective study with a complete follow-up in Chinese population; second, the implication of Chinese population in prediction of PE has not been closely examined by the PREDICTOR algorithm before; third, it supplements the data of combined multi-marker model to estimate patient-specific risks and investigates the performance of screening for two placental-related conditions; fourth, our screening approach effectively tests the feasibility of its future large-scale implementation in China and involves health economic considerations. As shown above in section of Methods in this report, maternal history recording and blood pressure measurement are a part of the normal routine pregnancy care. Also, maternal serum PAPP-A is widely used in screening for Down syndrome, and serum PLGF measurement can be further conducted on the same automated machines with same sample which can provide results quickly, with merely an additional cost for the reagents. This combining method for the prediction of PE can be incorporated into the present-day screening method for fetal aneuploidies, but whether or not this first-trimester screening method for PE could be readily available to the entire Chinese population requires further investigation.

Nonetheless, there are two important limitations of the study. One is that despite comparatively large sample size (over 3000), there were only eight cases of early PE due to the relatively low incidence of PE in southern China. But we evaluated the sample size by R package, and the results showed that for both early or late PE, the sample sizes were adequate and had enough power (> 99%). The prevalence of PE varies among different ethnic groups globally. Compared with the European and African American women, the incidence of PE in our research was relatively low [30]. The other limitation is that we did not perform the testing of Doppler ultrasound detection of UTPI. Our testing model did not include this marker (UTPI) into risk calculation, even though it has shown its promising value in predicting PE and SGA [31]. The reason is that measurement of UTPI requires specific training of sonographers to achieve uniformity of results among different operators. Due to large volume of outpatients, it is extremely difficult to ensure the quality of UTPI and to attain accurate results of every pregnancy as part of the current routine ultrasound scans in China. Presumably, the performance of our multivariate screening may be further improved by adding accurate UTPI, which is expected to be included in our next-step study. Yet it need be stressed that our screening model is still feasible in detection of PE with delivery < 34 weeks’ gestation.

The conventional approach to PE diagnosis is based on the occurrence of clinical symptoms discovered during routine obstetrics clinical visit in the second or third trimester of pregnancy, however, a new pyramid of pregnancy care having been proposed in 2011 states that effective early identification of the high-risk groups of subsequently developing PE and other pregnancy complications in the first trimester could reduce the incidence of these complications through pharmacological intervention with low-dose aspirin (LDA) [32, 33]. Further evidences in European populations have suggested that the risk of PE could be predicted and greatly reduced by the prophylactic application of LDA [34]. The aforementioned findings have demonstrated the vital role the prediction of PE plays in its clinical diagnosis, prevention and incidence reduction. What’s more, the ASPRE trial (Combined Multi-Marker Screening and Randomized Patient Treatment with Aspirin for Evidence-Based Preeclampsia Prevention) has shown that 150 mg/day of aspirin taken from 11 to 14 weeks to 36 weeks of gestation had reduced the incidence of early and preterm PE [35]. A secondary analysis of data from the ASPRE trial reported that in pregnancies at high risk of PE, treatment with LDA has shorten the length of stay in the neonatal intensive care unit [36]. Another research deriving data from SPREE (Screening programme for preeclampsia) and ASPRE concluded that the combined screening test of first-trimester pregnancies for PE could also identify a high proportion of cases of preterm SGA which can be prophylactically prevented by the use of aspirin [37]. But only a few small-sample studies in China have reported that pregnant women with high risk of PE obtained a better maternal and child prognosis after preventively taking aspirin [38]. Li et al. has launched a multicenter randomized controlled trial (RCT), low-dose Aspirin in the Prevention of Pre-Eclampsia in China (APPEC study), to evaluate the efficacy of LDA for PE prevention on high-risk pregnancies in Chinese [39]. Nevertheless, to achieve this, researches of accurate PE prediction at 11–13 weeks in Chinese are pivotal to future RCTs in the determination of the role that LDA plays in reducing the incidence of PE.

Our first-trimester integrated screening is valid in terms of predicting early PE, but late-onset PE (i.e. ≥34 weeks) accounts for a large proportion of all PE, which remains a significant challenge of effective early screening. Furthermore, the performance of prediction for isolated SGA is not as good as the one for SGA with PE. Consequently, screening strategy for late PE and isolated SGA demands additional potential markers and more effective screening model.

## Conclusions

To conclude, this study shows that our screening model could be effective for predicting early PE in Chinese pregnancies even without requiring ultrasound UTPI, but it is relatively less valuable for late PE and SGA neonates in the absence of PE. Furthermore, the performance of SGA screening by same protocol is strongly associated with PE.

## Additional file

Additional file 1: Study data. This data sheet includes the testing results of markers and prediction values of PE by PREDITCTOR software of all the participants in this study. (XLS 1417 kb)

## Data Availability

All data generated or analyzed during this study are available from the corresponding author on reasonable request.

## Abbreviations

ACOG: American Congress of Obstetricians and Gynecologists
APPEC: Low-dose Aspirin in the Prevention of Pre-Eclampsia in China
ASPRE: Combined Multi-marker Screening and Randomized Patient Treatment with Aspirin for Evidence-Based Preeclampsia Prevention
AUC: Area under the receiver operating characteristics curves
BMI: Body mass index
CRL: Crown rump length
DR: Detection rate
FMF: Fetal Medicine Foundation
FPR: False positive rate
GA: Gestation age
IQR: Interquartile range
IVF: In vitro fertilization
LDA: Low-dose aspirin
MAP: Mean arterial pressure
MoM: Multiple of median
NICE: National Institute for Health and Clinical Excellence
PAPP-A: Pregnancy associated plasma protein A
PE: Preeclampsia
PLGF: Placental growth factor
PPV: Positive predictive value
RCT: Randomized Controlled Trial
ROC: Receiver operating characteristics
SGA: Small-for-gestational-age
SPREE: Screening programme for pre-eclampsia
UTPI: Uterine artery pulsatility index
